# (‐)‐Epigallocatechin gallate‐loaded polycaprolactone scaffolds fabricated using a 3D integrated moulding method alleviate immune stress and induce neurogenesis

**DOI:** 10.1111/cpr.12730

**Published:** 2019-11-20

**Authors:** Yun Qian, Zhixiao Yao, Xu Wang, Yuan Cheng, Zhiwei Fang, Wei‐En Yuan, Cunyi Fan, Yuanming Ouyang

**Affiliations:** ^1^ Department of Orthopedics Shanghai Jiao Tong University Affiliated Sixth People’s Hospital Shanghai China; ^2^ Shanghai Sixth People’s Hospital East Affiliated to Shanghai University of Medicine & Health Sciences Shanghai China; ^3^ Engineering Research Center of Cell & Therapeutic Antibody Ministry of Education, and School of Pharmacy Shanghai Jiao Tong University Shanghai China

**Keywords:** (‐)‐epigallocatechin gallate, immune milieu, integrated moulding, nerve scaffold

## Abstract

**Objectives:**

In peripheral neuropathy, the underlying mechanisms of nerve and muscle degeneration include chronic inflammation and oxidative stress in fibrotic tissues. (‐)‐Epigallocatechin gallate (EGCG) is a major, active component in green tea and may scavenge free radical oxygen and attenuate inflammation. Conservative treatments such as steroid injection only deal with early, asymptomatic, peripheral neuropathy. In contrast, neurolysis and nerve conduit implantation work effectively for treating advanced stages.

**Materials and methods:**

An EGCG‐loaded polycaprolactone (PCL) porous scaffold was fabricated using an integrated moulding method. We evaluated proliferative, oxidative and inflammatory activity of rat Schwann cells (RSCs) and rat skeletal muscle cells (RSMCs) cultured on different scaffolds in vitro. In a rat radiation injury model, we assessed the morphological, electrophysiological and functional performance of regenerated sciatic nerves and gastrocnemius muscles, as well as oxidative stress and inflammation state.

**Results:**

RSCs and RSMCs exhibited higher proliferative, anti‐oxidant and anti‐inflammatory states in an EGCG/PCL scaffold. In vivo studies showed improved nerve and muscle recovery in the EGCG/PCL group, with increased nerve myelination and muscle fibre proliferation and reduced macrophage infiltration, lipid peroxidation, inflammation and oxidative stress indicators.

**Conclusions:**

The EGCG‐modified PCL porous nerve scaffold alleviates cellular oxidative stress and repairs peripheral nerve and muscle structure in rats. It attenuates oxidative stress and inflammation in vivo and may provide further insights into peripheral nerve repair in the future.

## 1 INTRODUCTION

Radiation treatment results in some inevitable side effects and can cause mild or severe damage to patients. In long‐term follow‐up, peripheral neuropathy can occur as a late complication induced by radiation, even though peripheral nerves are well‐differentiated tissues and are relatively insensitive to radiation.^1^ Associated numbness and pain significantly affect patient quality of life. Steroid injections alleviate early asymptomatic neuropathy, but cannot treat advanced cases,^2^ while surgical interventions for nerve release have proven to be very helpful in preclinical and clinical scenarios of severe peripheral neuropathy.^3^ In a radiation‐induced peripheral neuropathy model using rats, a chitosan nerve scaffold successfully improved functional nerve recovery and restored nerve structures as evaluated by magnetic resonance imaging.^4^ Grooved silica conduits have been used for repairing short sciatic nerve gaps in rats.^5^ Poly(dl‐lactide‐epsilon‐caprolactone) nerve bridges resulted in satisfactory nerve recovery across a 10 mm nerve defect compared with autografts.^6^ A polyglycolic acid/collagen nerve scaffold filled with lamina was found to contribute to the regeneration of an 80 mm nerve defect in common peroneal nerves in dogs.^7^ Clinically, nerve release operations such as mentoplasty contribute to the alleviation of symptomatic neuropathy.^8^

The underlying pathophysiological changes caused by radiation‐induced peripheral neuropathy mainly involve electrophysiological and histological alterations, chronic inflammatory reactions and oxidative stress responses in active fibrosis. Fibrotic tissues cause severe entrapment of peripheral nerves and lead to prominent muscle atrophy. Endplate muscle degeneration and muscle strength decrease are major complications of peripheral neuropathy which significantly reduce patient quality of life.^9^ In peripheral neuropathy, large quantities of nitric oxide synthase (NOS) are synthesized, resulting in the massive death of injured tissues, including Schwann cells and skeletal muscle cells. Meanwhile, pro‐inflammatory cytokines such as tumour necrosis factor‐α (TNF‐α) and interleukin‐6 (IL‐6) increase significantly and impair nerve and muscle function. These cytokines are induced by the continuous existence of macrophages, which initially clear myelin debris and later destroy the microenvironment for nerve and muscle regeneration due to the release and accumulation of various cytokines.^10^ The transcription factor NF‐E2‐related factor (Nrf2)/anti‐oxidant response element (ARE) signalling pathway can regulate the balance of oxidative stress in the nervous system. Improved expression of Nrf2 is important for repairing nerve structure and functions by inhibiting oxidative nerve damage during peripheral nerve regeneration.^11^

Green tea has gained wide attention around the world for its attractive aroma and rich benefits,^12^ and is rich in polyphenols, including flavonoids and catechins, which play a key role in scavenging free radical oxygen in the human body. Among its many polyphenolic compounds, (‐)‐epigallocatechin gallate (EGCG) is the most effective free radical oxygen scavenger.^13^ In a peripheral neuropathy model, Wei et al reported that EGCG could attenuate oxidative stress in motor neurons at dosages of 25 or 50 mg/kg.^14^ In addition, EGCG could induce an Nrf2‐dependent anti‐oxidant response and clear reactive oxygen species (ROS) in human epithelial cells.^15^ Therefore, we aimed to evaluate the potential influence of EGCG on oxidative stress and inflammation in radiation‐induced peripheral neuropathy, which is a poorly studied topic. Daily injection has many shortcomings, such as operational redundancy and inaccurate disease site positioning. Instead, a controlled style mediated by a scaffold facilitates gradual drug release into regional diseased tissues and helps improve long‐term recovery. In this study, an EGCG polycaprolactone (PCL) scaffold was designed in a controlled release style. PCL is a common synthetic material for manufacturing nerve scaffolds. It has many important characteristics, including a suitable degradation rate, biocompatibility and mechanical stability, all of which should be considered when selecting appropriate scaffold materials.^16^, ^17^, ^18^, ^19^, ^20^ PCL is very useful and advantageous in long‐term peripheral nerve regeneration due to its relatively low degradation speed. It does not collapse after a short period of time or cause nerve contraction.^21^ Laminin nanofibre‐mixed PCL electrospun scaffolds increased neuron‐like PC12 cell proliferation and adhesion.^22^ Gold nanoparticle‐loaded PCL conduits improved the regenerative capacity of nerve injuries.^23^, ^24^ Graphene scaffolds also induced angiogenesis and stimulated neurite extension by improving Schwann cell viability.^25^, ^26^ In the present study, we investigated the anti‐oxidant and anti‐inflammatory roles and impacts of an EGCG‐loaded PCL scaffold on Schwann and skeletal muscle cell proliferation in vitro. Moreover, we further evaluated its contributions to functional sciatic nerve recovery and skeletal muscle restoration, as well as in vivo clearance of oxidative stress and attenuation of inflammation.

## 2 MATERIALS AND METHODS

### 2.1 Fabrication and characterization of EGCG‐loaded PCL scaffolds

EGCG and PCL (relative molecular weight: 6000) were purchased from Sigma‐Aldrich (USA). The EGCG powder was mixed with PCL solution before sonication for ten minutes. Then, the mixture was injected into a tubular mould followed by jet spraying using a rotating sprayer. The air was compressed to spray the solution from a collection container. Afterwards, the EGCG/PCL membrane was created via dichloromethane evaporation. After the first layer (the innermost layer was solid), the middle and outermost layers were sprayed. This was the integrated moulding method for multiple‐layered EGCG/PCL conduit fabrication. In the meantime, the microneedles were used to create various pores on the surface of the EGCG/PCL conduit. They were in excellent alignment and uniform in size. The microneedles were removed after the complex conduit solidified. The same procedure was applied to PCL conduit preparation.

The EGCG/PCL conduit was evaluated using a scanning electron microscope (Sirion 200, USA) for surface structure morphology. Gold coating was sprayed on all samples, and samples were observed at 5 kV. Random images were selected, including those magnified 5000× and 20000×. We measured the conduit wall thickness, elongation at break and surface elastic modulus using the Vernier calliper and a nanoindenter machine (G200, Agilent, Santa Clara, USA). Fourier‐transform infrared spectroscopy (FTIR) was performed to evaluate the physical interaction between EGCG and PCL to show the successful fabrication of an EGCG‐modified PCL conduit.

(‐)‐Epigallocatechin gallate release from the EGCG/PCL conduit in vitro was monitored at a maximum absorption wavelength of 276 nm using the Multi‐Mode Microplate Reader (Thermo Fisher Scientific, Carlsbad, CA, USA). Standard drug concentration curves were constructed at specific intervals using linear regression. Conduit samples were incubated in phosphate buffer saline (PBS; Thermo Fisher Scientific, Carlsbad, CA, USA) at 37°C. For each sample, 5 mL PBS was removed and replaced with fresh buffer at predetermined time intervals. The ultraviolet absorbance of EGCG was measured and converted to the appropriate concentration according to the established standard curves. Finally, the cumulative drug release graph was plotted via GraphPad 5.

### 2.2 Cell culture

Rat Schwann cells (RSCs) and rat skeletal muscle cells (RSMCs) were provided by the cell bank of the Chinese Academy of Sciences (Shanghai, China). RSCs and RSMCs were cultured in a humidified 37°C CO_2_ incubator. We seeded 1 × 10^5^ RSCs and 1 × 10^5^ RSMCs in EGCG/PCL and PCL scaffolds the size of 24‐well plates, respectively. The culture medium included high‐glucose Dulbecco's modified Eagle's medium supplemented with 10% foetal bovine serum (Gibco, USA) and 1% penicillin/streptomycin solution (Gibco, USA). Both RSCs and RSMCs were treated with the oxidative stimulator H_2_O_2_ (50 μmol/L) for 30 minutes before evaluating the levels of ROS and inflammation.

### 2.3 Cell counting kit 8 (CCK8) assay

CCK8 was used to analyse the proliferation of RSCs and RSMCs from EGCG‐loaded PCL and PCL scaffolds at 24, 72, 120 and 168 hours. The culture medium was refreshed every two days. A CCK8 working solution was added to the RSCs and RSMCs and co‐cultured in 5% CO_2_. Medium (100 μL) from each well was analysed by a spectrophotometer at 450 nm.

### 2.4 Immunofluorescence assay

Cellular debris of RSCs from different scaffolds was washed using PBS, fixed with 4% paraformaldehyde (PFA) solution for 20 minutes at 25°C and immersed in Triton working solution (Thermo Fisher Scientific, Carlsbad, CA, USA) for 10 minutes, and then blocked with bovine serum albumin. We incubated samples with different primary antibodies (Thermo Fisher Scientific, Carlsbad, CA, USA) for 12 hours at 0°C and a secondary antibody for 3 hours at 25°C. Nuclei were stained with diamidino‐phenylindole (DAPI) (1:500). The primary antibodies included anti‐Ki‐67 (1:400), anti‐S100α (1:250) and anti‐Nrf2 (1:150). Alexa Fluor 488‐conjugated IgG (1:250; Thermo Fisher Scientific, Carlsbad, CA, USA) was used as the secondary antibody. The slides were observed under a confocal microscope (Leica/TCS SP8 STED 3X, Wetzlar, Germany).

### 2.5 Western blotting (WB) and quantitative polymerase chain reaction (qPCR)

For evaluation of oxidative stress in vitro, representative markers such as Nrf2, manganese superoxide dismutase (MnSOD), heme oxygenase‐1 (HO‐1) and glutamate‐cysteine ligase catalytic (GCLC) were used in this study. Protein (20 μg) was loaded on 10% sodium dodecyl sulphate polyacrylamide gel, electrophoresed and immediately transferred to nitrocellulose sheets for 3 hours. All the samples were transferred to poly(vinylidene fluoride) membranes and incubated at 4°C overnight. Primary antibodies (Cell Signaling Technology, CST, USA) were added in the following order: Nrf2 (1:1500), MnSOD (1:1500), HO‐1 (1:1500) and GCLC (1:1500). β‐Actin (1:4000) served as the control. Densitometry was used for estimating different bands via ImageJ software. Total RNA was collected from RSCs and RSMCs using TRIzol reagent (Gibco, USA) according to the manufacturer's protocol. First, RSCs and RSMCs were lysed and the lysate and isolated RNA were collected, followed by first‐strand DNA synthesis. Ct values and relative quantities were calculated via the RT‐PCR machine programme by comparing the Ct of all samples with the standard curve's arbitrary values. The gene expression was compared with GAPDH. Primer sequences are displayed in Table S1.

### 2.6 ROS and mitochondrial membrane potential evaluation

The oxidative stimulator H_2_O_2_ induced intracellular ROS as determined by 2’, 7’‐dichlorofluorescein diacetate (DCFDA) staining (Beyotime, Shanghai, China). DCFDA is a nonfluorescent polar probe. Cells were cultured in EGCG/PCL and PCL scaffolds for three days before the experiment, incubated with DCFDA working liquid for 30 minutes at 37°C and then washed three times with Hank's Balanced Salt Solution to remove any remaining uncombined solution. Finally, the prepared samples were ready for flow cytometry and JC‐1 staining for mitochondrial membrane potential measurement. Cells were cultured in media supplemented with JC‐1 liquid (1 μg/mL, Thermo Fisher Scientific, Carlsbad, CA, USA) for 30 minutes, washed three times with PBS and evaluated by fluorescence microscopy.

### 2.7 Animal surgery

All Sprague Dawley rats (male, 150 g) were kept in a pathogen‐free room and had free access to water and food. All rats received 40‐Gy radiation to the right thigh (approximately 15 mm diameter) to create the peripheral neuropathy model. Other tissues were properly covered with thick lead bricks during radiation exposure. Five rats died from infection and haemorrhage after radiation therapy. The remaining 30 animals were randomly grouped as follows: EGCG/PCL, PCL and sham groups (10 rats per group with two time points). Pentobarbital sodium (60 mg/kg) was injected intraperitoneally for anaesthesia. An incision was made, in sterile conditions, on the right side to expose the sciatic nerves. Neurolysis was performed on all rats, and the right sciatic nerve was encapsulated immediately with the EGCG/PCL or PCL conduit. Rats in the sham group did not receive neurolysis or conduit encapsulation. Muscle and skin were carefully sutured followed by injection with 10^5^ units of penicillin for anti‐bacterial prophylaxis. Postsurgical observation was performed at 6 and 12 weeks. Animal welfare was ensured according to the protocol issued by the Institutional Animal Care and Use Committee of Shanghai Jiao Tong University (SJTU, No. A2017073).

### 2.8 Walking track analysis and electromyograph

We included walking track analysis for functional evaluation. In a quiet atmosphere, footprints were evaluated as follows: the Sciatic Function Index (SFI) was measured based on three parameters, including intermediate toe length, toe spread and footprint length. The SFI value ranged from ‐100 to 0.

A bipolar needle electrode was implemented in this experiment. A recording electrode and corresponding reference electrode were placed on the distal muscle and proximal nerve side, respectively. Before recording, rats were administered anaesthesia at the same dosage used for neurolysis and conduit implantation. We recorded and evaluated nerve conducting velocity (NCV) and distal compound action potential (DCMAP) from three experimental groups.

### 2.9 Transmission electron microscopy and immunohistochemistry

The middle 15 mm regenerated nerves and gastrocnemius muscle were harvested at 6 and 12 weeks after conduit implantation. Haematoxylin and eosin (HE) and 1% toluidine blue (TB) staining were performed, then the nerve and muscle sections were fixed using paraformaldehyde working solution for 12 hours and then immersed in osmium tetroxide working and cacodylate buffer solutions. All samples were embedded and different staining methods were carried out, including HE and TB staining. The myelinated fibre count was calculated using a light microscope, and the samples were observed via transmission electron microscopy (TEM) to analyse areas of axon and myelin sheath thickness, using 4% uranylacetate and lead staining working solution for examination.

Regenerated sciatic nerves were separated into different sections to determine oxidant (nNOS), and anti‐oxidant levels (Nrf2 and MnSOD) as well as severity of inflammation (TNF‐α staining). The sections were fixed in paraformaldehyde working solution for 30 minutes, immersed in Triton working solution (Sigma‐Aldrich, Cambridge, USA) for 10 minutes, washed several times with PBS and incubated overnight at 4°C with primary antibodies: nNOS (1:150), Nrf2 (1:250), MnSOD (1:400) and TNF‐α (1:300), all purchased from Thermo Fisher Scientific, Carlsbad, CA, USA. Then, the biotinylated secondary antibody (1:200 Thermo Fisher Scientific, Carlsbad, CA, USA) was added to all samples for 1 hour at 25°C. Finally, the samples were incubated with streptavidin/ horseradish peroxidase (Dako, Santa Clara, USA). All sample slides were observed under a fluorescence or light microscope.

### 2.10 Muscle recovery evaluation

The right gastrocnemius muscle was dissected from all rats after nerve conduit implantation. Muscle mass was calculated to evaluate muscle atrophy. The Achilles tendon was dissected carefully, and the lower limb was connected with a muscle force measurement instrument (WPI, Shanghai, China). Contraction forces were provoked at 50 Hz for 5 seconds to the muscle and were calculated as a mean value. The evaluation was repeated three times. The final percentage of muscle strength was measured and compared with the contralateral healthy limb. In addition, muscle samples were kept in normal saline, homogenized and evaluated with the creatine phosphokinase (CK) activity kit (Sigma‐Aldrich, Cambridge, USA). Then, ultrathin gastrocnemius muscle sections from the irradiated sides were fixed with 4% PFA for 20 minutes followed by normal serum blocking. Morphological evaluation was carried out with HE and TB staining at 6 and 12 weeks postoperatively. We selected random fields and evaluated muscle fibres of different sections using Image‐Pro software.

### 2.11 Lipid peroxidation level (LPO) measurement

Lipid peroxidation level was evaluated by lipid peroxidase solution (Eagle Biosciences, Shanghai, China) in the regenerated sciatic nerve and gastrocnemius muscle. We added 650 μL working solution to 200 μL samples followed by the addition of 105 µL of 37% chlorine hydride to each tube. Samples were incubated at 45°C for 1 hour before centrifugation at 15 000 g for 15 minutes to obtain a clear supernatant. Finally, 3 × 150 µL of supernatant was transferred to a microplate and read at 586 nm (DU 640B; Beckman, Fullerton, CA, USA).

### 2.12 Macrophage infiltration

The presence and number of macrophages were assessed using CD68 protein as a marker via WB (1:1500) and immunohistochemistry (1:500) as described above. The relative quantitative analysis was dependent on ImageJ evaluation of the blot's grey value. The degree of macrophage infiltration was evaluated using CD68‐positive cell counting in the immunofluorescent assay by three independent observers.

### 2.13 Western Blotting

We digested and homogenized regenerated sciatic nerves that were homogenized in lysis buffer according to standard procedure. In brief, 20 μg protein was loaded on 10% sodium dodecyl sulphate polyacrylamide gel for electrophoresis and was immediately transferred to nitrocellulose sheets for 3 hours. All the samples were transferred to a poly (vinylidene fluoride) membrane and incubated at 4°C overnight. The primary antibodies (Thermo Fisher Scientific, Carlsbad, CA, USA) were anti‐IL‐6 (1:3000), anti‐TNF‐α (1:2500) and anti‐CD68 (1:1500). β‐Actin was used as the control. The relative quantitative analysis was dependent on ImageJ evaluation of the blot's grey values.

### 2.14 Statistics

All data are shown as mean ± standard deviation (SD) from at least three independent experiments. Data obtained in in vitro experiments were evaluated by an independent‐sample t test. Data from in vivo experiments were analysed by one‐way analysis of variance. *P* < .05 was considered significant. Statistics were performed using SPSS 19.0 (Chicago, IL, USA).

## 3 RESULTS

### 3.1 Fabrication and characterization of the EGCG/PCL scaffold

**Figure 1:**
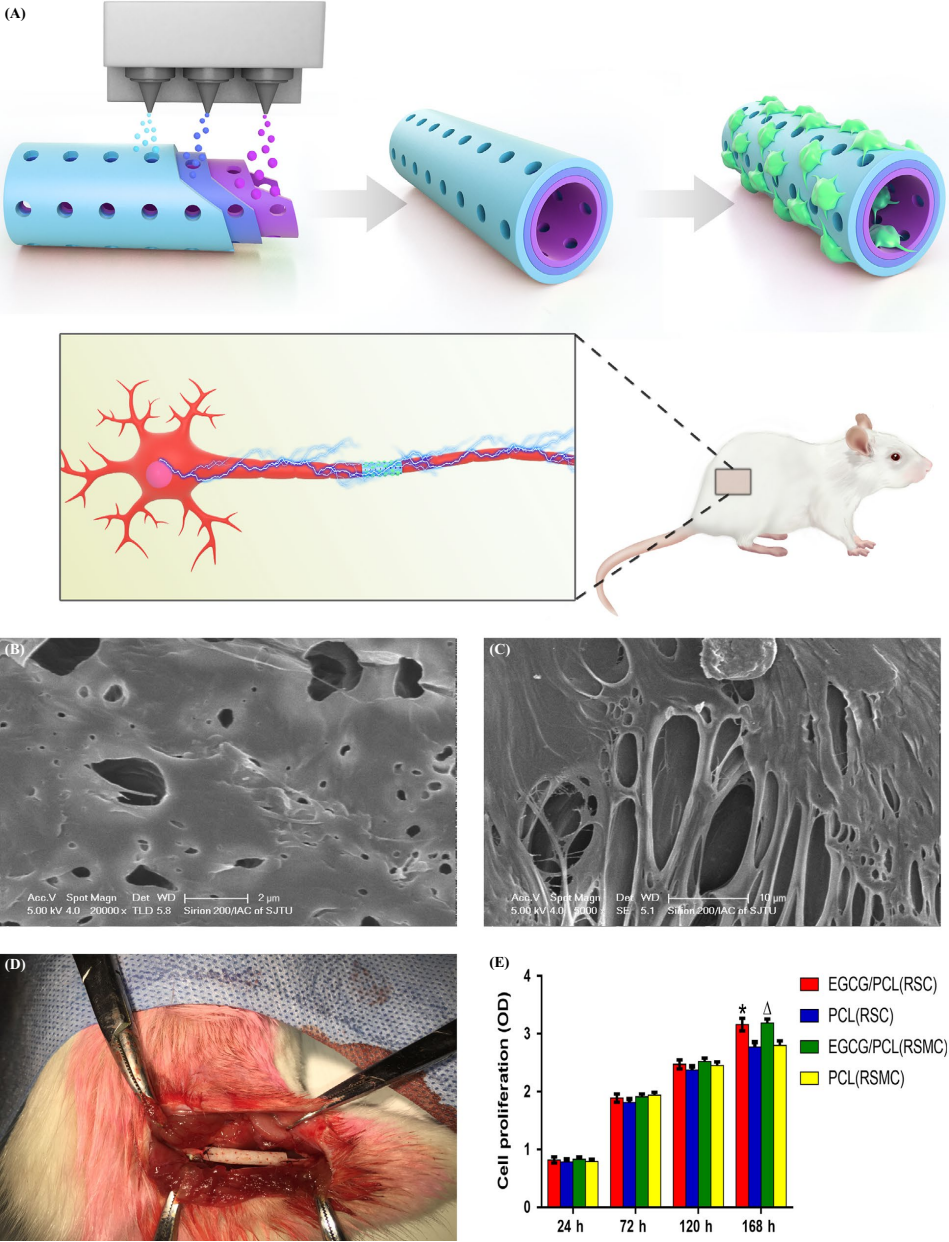
Scheme of (‐)‐epigallocatechin gallate‐loaded polycaprolactone scaffold fabrication using integrated moulding and nerve conduit implantation in rat models (A). Characterization of an (‐)‐epigallocatechin gallate‐loaded polycaprolactone nerve conduit. The scanning electron microscopy and optical images of the EGCG/PCL nerve conduit (B‐D). CCK8 assay for RSCs and RSMCs on EGCG/PCL and PCL scaffolds at 24, 72, 120 and 168 hours. *P < .05 compared with PCL scaffolds (RSCs), ^Δ^ *P* < .05 compared with PCL scaffolds (RSMCs) (E)

In this study, we fabricated an EGCG/PCL nerve conduit using the integrated moulding method (Figure 1A). We prepared a complex tubular mould that had concentric circles, consisting of the inner, outer and middle tubes. EGCG/PCL (1%) mixed solution was sprayed into the lumen of each tube mould. The same procedure was repeated twice to form a three‐layer porous structure. The wall thicknesses were 0.5 mm and 0.49 mm for the EGCG/PCL and PCL scaffolds, respectively. A microneedle that was previously prepared was added to the aligned pores (20 μm in diameter). At different magnifications, we identified the porous structure and stiff surface of this mixed material (Figure 1B,C) and also observed conduit implantation in the rat (Figure 1D). We then examined two mechanical properties of the EGCG/PCL and PCL scaffolds, the elongation at break and the elastic property. The elastic modulus of the EGCG/PCL scaffold was higher than that of the PCL scaffold. Furthermore, the addition of EGCG improved shear behaviour by increasing the elongation at break of the scaffold (Figure S1A,B). The results confirmed that the multiporous EGCG/PCL scaffold maintained a tubular shape and avoided compression of regenerated nerves. FTIR results showed that the peak value for EGCG was 3356 cm^‐1^, while the values for the PCL scaffolds were 1720 and 2942 cm^‐1^ and those for the EGCG/PCL scaffolds were 2948, 1728 and 3357 cm^‐1^, indicating the successful fabrication of a combined conduit due to a simple physical mixture without a chemical interaction between EGCG and PCL (Figure S2A‐C). Based on the above results, the EGCG/PCL nerve conduit displayed a multilayered and porous structure as well as excellent mechanical support for long‐term nerve restoration.

**Figure 2:**
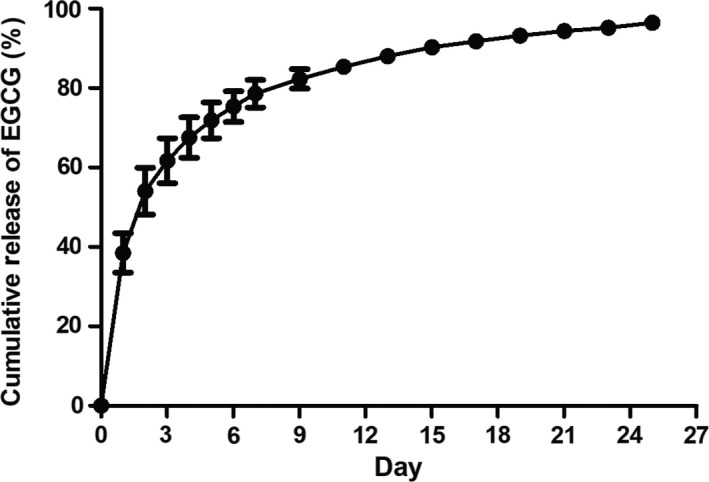
In vitro cumulative release of EGCG from EGCG/PCL conduits in PBS by ultraviolet‐visible spectrometry

We evaluated the release of EGCG from the conduit and confirmed a controlled release pattern. For the EGCG/PCL conduit, no sudden drug release was observed during the first 24 hours (Figure 2). Maximum release was obtained at day 30, due to the excellent integrated fabrication. These results indicate that the EGCG/PCL conduit released EGCG at a sustained rate that facilitated steady and persistent EGCG release for long‐term in vivo regeneration.

### 3.2 Effects of the EGCG/PCL scaffold on cell proliferation and specificity

**Figure 3:**
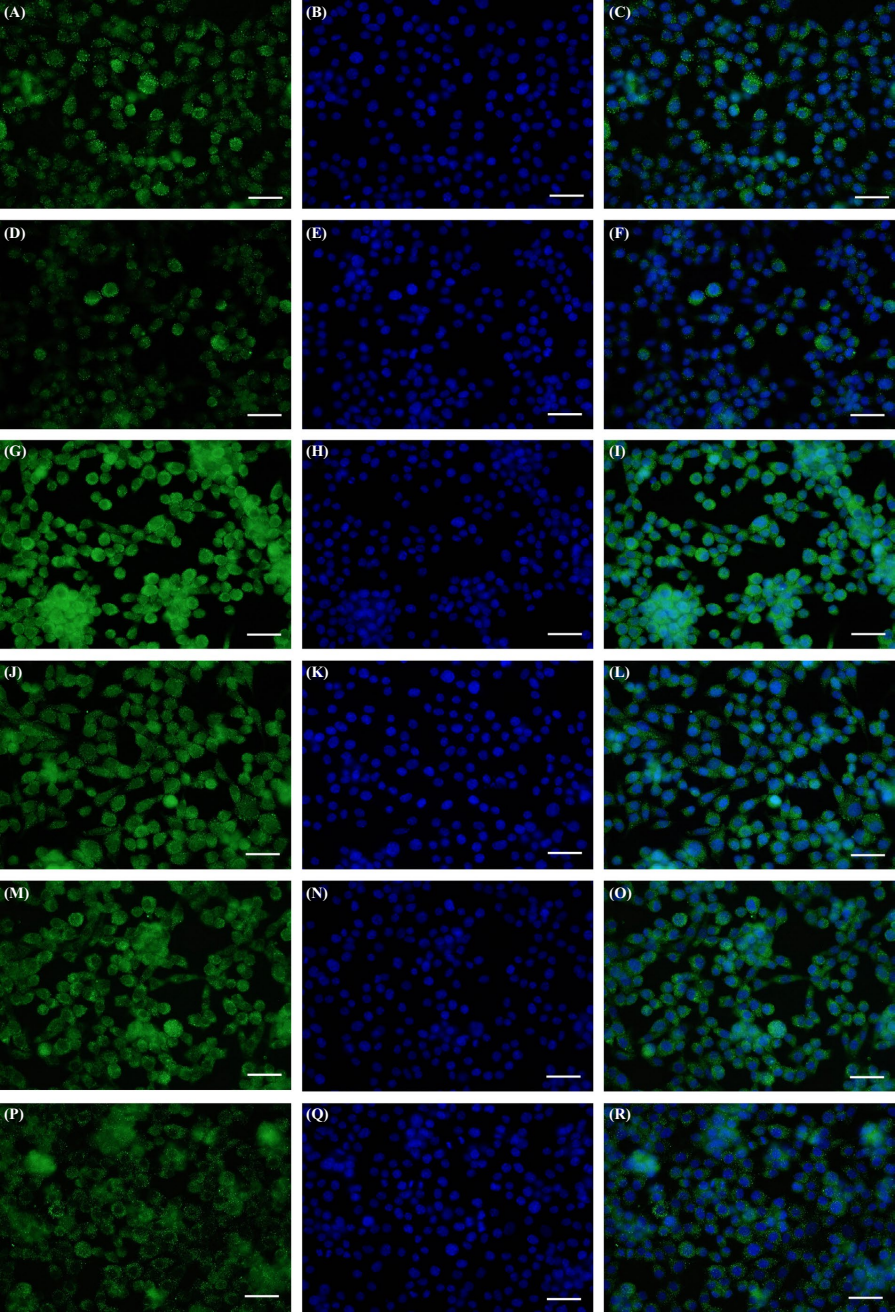
Ki‐67 immunofluorescent staining for RSCs on EGCG/PCL scaffolds (A, B, C) and on PCL scaffolds (D, E, F). S100 immunofluorescent staining for RSCs on EGCG/PCL scaffolds (G, H, I) and PCL scaffolds (J, K, L). Nrf2 immunofluorescent staining for RSCs on EGCG/PCL scaffolds (M, N, O) and PCL scaffolds (P, Q, R). A, D, G, J, M and P show specific marker staining (in detail, A & D: Ki‐67, G & J: S100, M & P: Nrf2). B, E, H, K, N and Q show nuclei staining. C, F, I, L, O and R show merged images of marker and nuclei staining. The scale bar is 100 μm

The results of 24‐, 72‐ and 120‐hour proliferation assays all showed that cells had a similarly high proliferative capacity on the two scaffolds. After 168 hours, both RSCs and RSMCs showed significantly higher proliferation on the EGCG/PCL scaffold than the control scaffold (*P* < .05, Figure 1E). The proliferative ability of the EGCG/PCL scaffold was further validated by WB and qPCR analysis using two growth‐related markers, Ki‐67 and GAP‐43. Ki‐67, a nuclear protein, plays a vital role in cellular proliferation and exists actively in the G1, S and G2 phases of mitosis. GAP‐43 is a membrane‐associated phosphorylated protein and is closely related to neurite outgrowth and plasticity. WB and qPCR showed that Ki‐67 expression was significantly elevated on the EGCG/PCL scaffold compared with the PCL scaffold, by 2.4‐fold for RSCs and 2.0‐fold for RSMCs (*P* < .05) (Figure S3A). It was also confirmed by immunofluorescence analysis that the EGCG/PCL scaffold could stimulate RSC proliferation more discernably than the PCL scaffold (Figure 3A‐F). Meanwhile, qPCR showed that GAP‐43 also increased by 2.7‐fold for RSCs and by 2.3‐fold for RSMCs on the EGCG/PCL scaffold compared with the PCL counterpart (Figure S3B). In addition, S100 expression levels from the EGCG/PCL group increased significantly compared with the PCL scaffold, for RSCs (Figure 3G‐L). These results show that the EGCG‐based polymeric scaffold had a beneficial effect on RSC and RSMC growth and maintenance of RSC specificity.

### 3.3 Effects of the EGCG/PCL scaffold on ROS clearance

Nrf2, MnSOD, HO‐1 and GCLC levels were evaluated to assess ROS status. Immunofluorescence showed that Nrf2 expression in RSCs was significantly increased on the EGCG/PCL scaffold in contrast to the PCL scaffold (Figure 3M‐R). We showed that Nrf2 expression increased by 7.4‐fold in RSCs and by 6.8‐fold in RSMCs on the EGCG/PCL scaffold compared with the PCL scaffold (*P* < .05) (Figure S3C). As compared to the levels on the PCL scaffold, the MnSOD, HO‐1 and GCLC expression levels on the EGCG/PCL scaffolds were 2.8‐, 2.3‐ and 3.1‐fold higher for RSCs and 2.4‐, 2.8‐ and 3.3‐fold higher for RSMCs, respectively (*P* < .05, Figure S3D‐F).

In addition, intracellular ROS levels were assessed using the DCFDA assay. The result showed that EGCG treatment significantly reduced ROS levels in RSCs and RSMCs, as indicated by flow cytometry (Figure S4A‐D,I). Moreover, the mitochondrial membrane potential was evaluated using JC‐1 staining. The pictures showed that the EGCG/PCL scaffold had a stronger fluorescence intensity than the control group, indicating a more efficient mitochondrial membrane potential preservation (Figure S4E‐H,J). These results show that EGCG could improve clearance of oxidative stress in both RSCs and RSMCs.

### 3.4 Effects of the EGCG/PCL conduit on functional and electrophysiological performance

No obvious complications such as ulcers, infections or wound healing failures were noticed at 6 and 12 weeks after conduit implantation. At the first two time points, the regeneration rate was obviously higher in the EGCG/PCL than the PCL (n = 5, *P* < .05), and sham (n = 5, *P* < .05) groups based on SFIs (Figure S5A). For electrophysiological analysis, NCV in EGCG/PCL conduit group was significantly upregulated in comparison with the PCL and sham counterparts (*P* < .05) at both 6 and 12 weeks (Figure S5B). The DCMAP value was similarly higher in the EGCG/PCL than the PCL and sham counterparts (*P* < .05, Figure S5C). This indicated that the EGCG/PCL conduit improved the motor function of the hindlimb and restored electrical transduction in injured nerves. The EGCG/PCL conduit contributed to functional and electrophysiological recovery after a long‐term in vivo process.

### 3.5 Effects of the EGCG/PCL conduit on morphological nerve regeneration

The 15 mm middle nerve sections were removed from experimental rats to evaluate the myelinated axons and overall nerve regeneration. HE and TB staining showed that myelinated axon number, myelin sheath thickness, area of axon regeneration and average diameter of remyelinated axons were all significantly improved in the EGCG/PCL conduit in comparison with the PCL and sham groups (*P* < .05) (Figure S5D‐E, & S6A‐F). The regenerated axon area also increased significantly in the EGCG/PCL group, showing a notable difference compared with the PCL and sham groups (*P* < .05, Figure S5F & S6A‐F). Myelin sheath thickness was further evaluated using TEM and was found to have the greatest increase in the EGCG/PCL conduit group, followed by the PCL and sham groups (*P* < .05, Figure S5G & S6G‐O).

### 3.6 Effects of the EGCG/PCL conduit on oxidative stress

**Figure 4:**
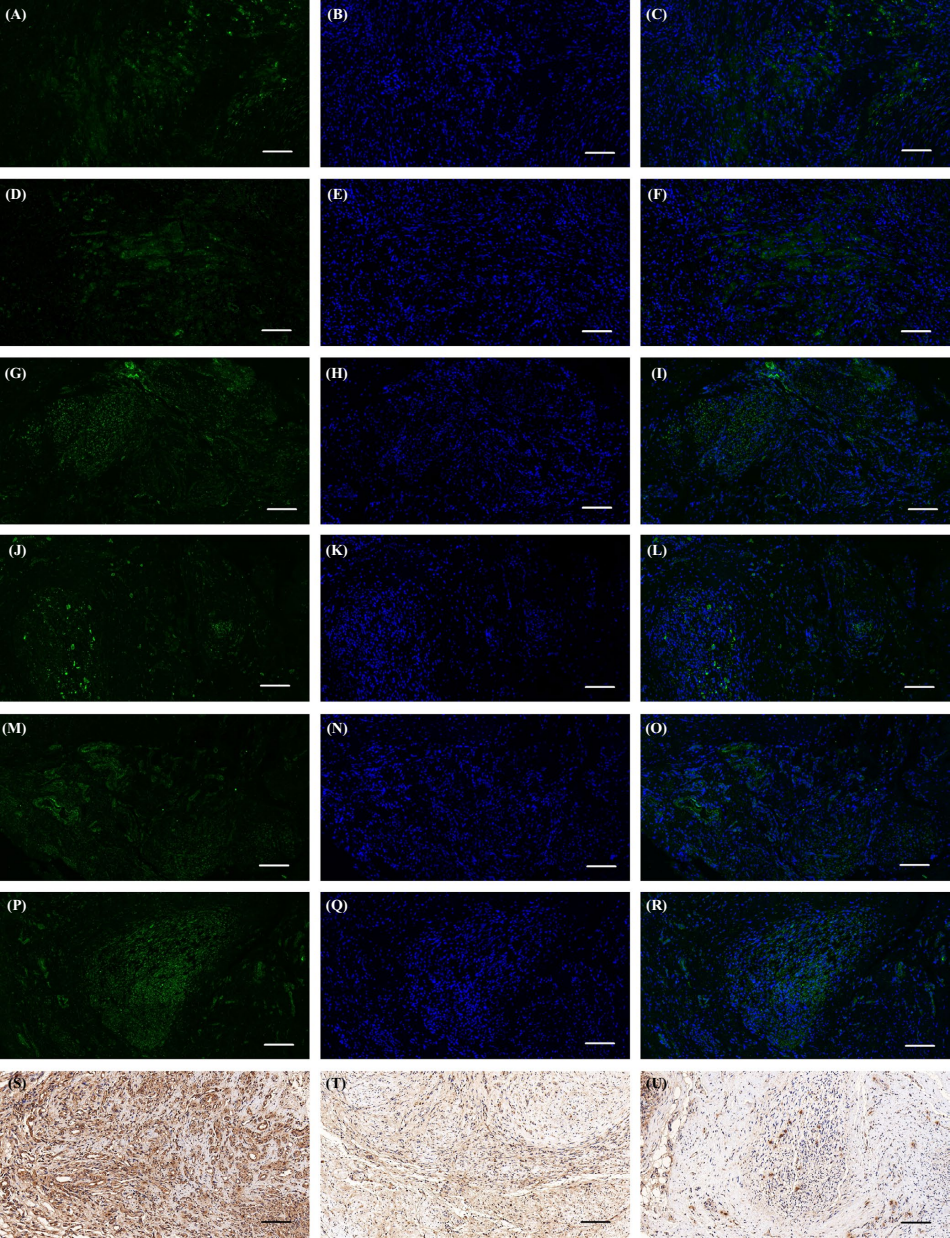
nNOS (A‐I), Nrf2 (J‐R) and MnSOD (S‐U) immunohistochemistry staining for three groups: EGCG/PCL conduit group (A‐C, J‐L, S), PCL conduit group (D‐F, M‐O, T) and sham group (G‐I, P‐R, U). The scale bar is 100 μm

**Figure 5:**
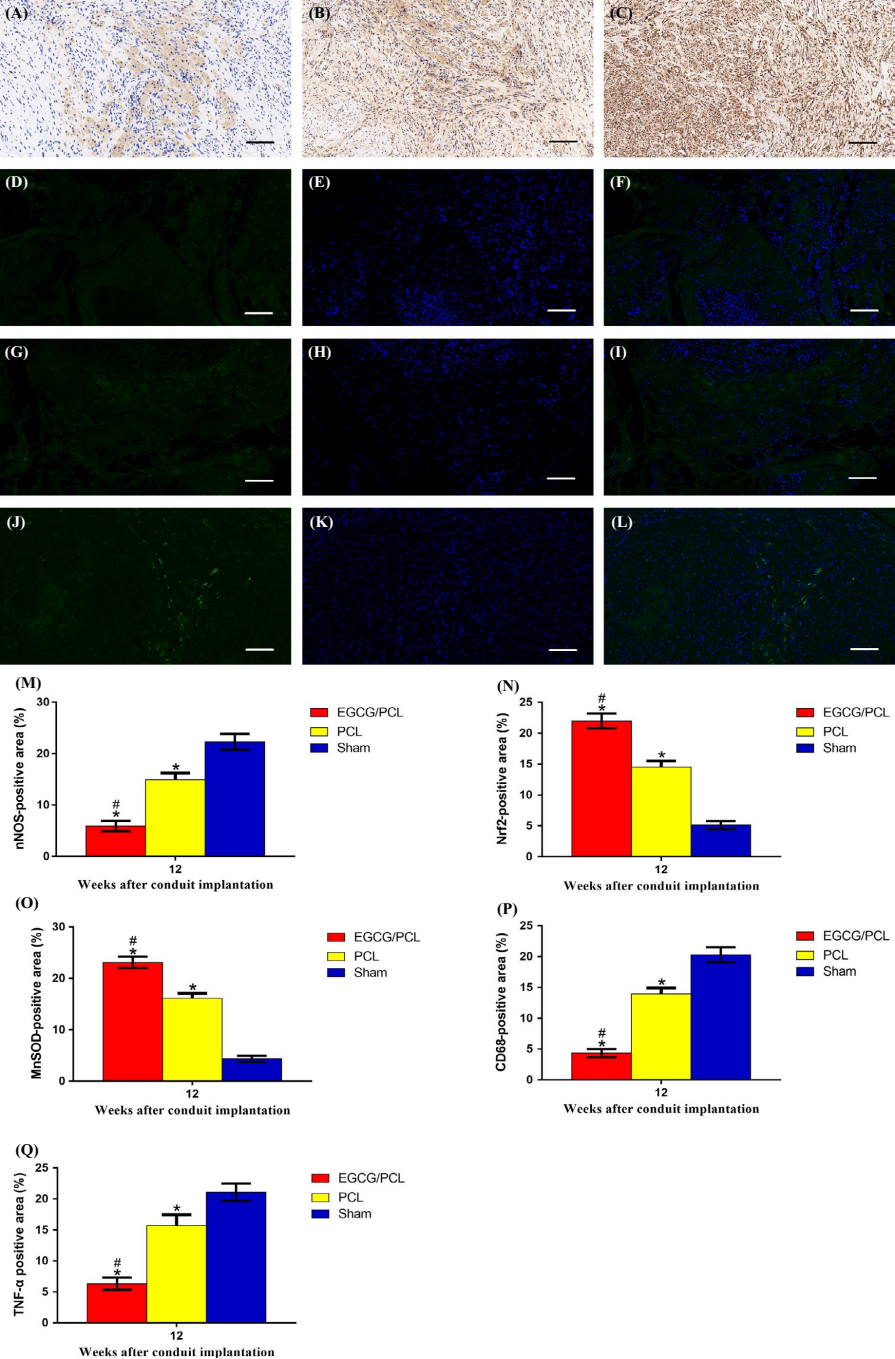
CD68 (A‐C) and TNF‐α (D‐L) immunohistochemistry staining for three groups: EGCG/PCL conduit group (A, D‐F), PCL conduit group (B, G‐I) and sham group (C, J‐L). The scale bar is 100 μm. nNOS‐positive area (%) (M), Nrf2‐positive area (%) (N), MnSOD‐positive area (%) (O), CD68‐positive area (%) (P)and TNF‐α–positive area (%) (Q). *P < .05 compared with sham group; #*P* < .05 compared with PCL conduit group

The oxidative stress level was further analysed in vivo using a specific oxidative stress marker, nNOS, as well as anti‐oxidant markers Nrf2 and MnSOD. The nNOS level was barely detectable in the EGCG/PCL conduit group but was noticeably expressed in the PCL and sham groups (*P* < .05, Figure 4A‐I & 5M). The results for Nrf2 and MnSOD were contradictory, with a marked increase in the EGCG/PCL conduit group compared with the PCL and sham groups (*P* < .05, Figure 4J‐U & 5N, O). In addition, we evaluated LPO, a common indicator for oxidative stress, in regenerated nerve and gastrocnemius muscle samples, and compared the results in all groups at two time points. The results showed that the LPO level was highest in the sham conduit followed by the PCL conduit (*P* < .05) and lowest in the EGCG/PCL group (*P* < .05, Figure S5H,I). The marked decrease in nNOS and LPO levels, along with increased Nrf2 and MnSOD expression, all indicate the anti‐oxidant ability of EGCG in radiation‐induced peripheral neuropathy.

### 3.7 Effects of the EGCG/PCL conduit on chronic inflammation

To evaluate tissue inflammation, we evaluated macrophage presence in the tissue. Macrophage infiltration indicated lasting inflammation and other resulting physiological changes. CD68 expression, a common marker of the macrophage lineage, was evaluated in the EGCG/PCL, the PCL and sham groups. The number of CD68‐positive cells increased significantly in nerve tissues from the PCL conduit and sham groups but decreased significantly in the EGCG/PCL conduit group (*P* < .05, Figure 5A‐C,P). From the staining of CD68‐positive cells, we concluded that EGCG effectively reduced macrophage infiltration in the long‐term regeneration of peripheral nerves.

**Figure 6:**
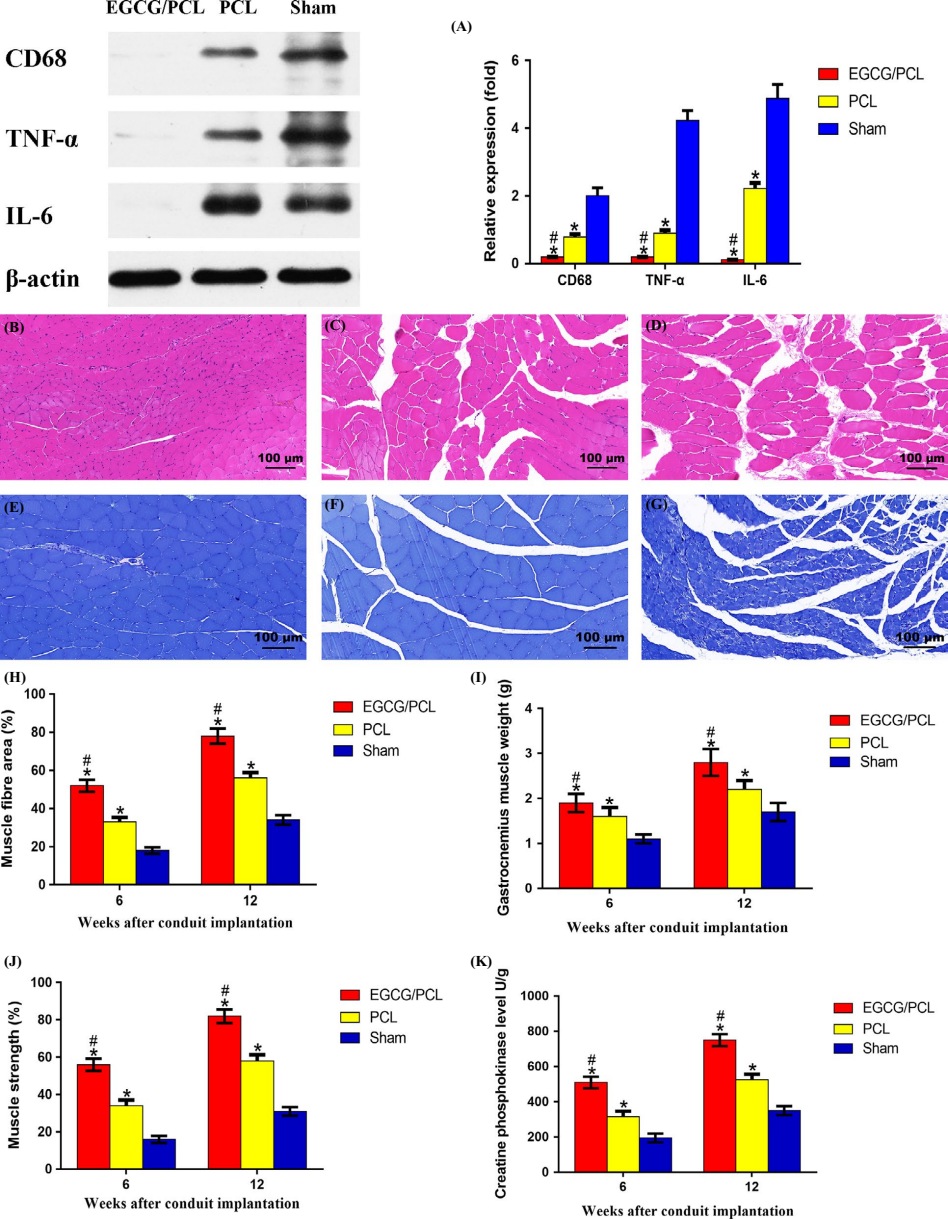
WB for CD68, TNF‐α and IL‐6 among three groups and their relative expression (A). Gastrocnemius muscle morphology evaluation from three groups. HE staining for the EGCG/PCL conduit group (B), PCL conduit group (C) and sham group (D), and TB staining for the EGCG/PCL conduit group (E), PCL conduit group (F) and sham group (G). The scale bar is 100 μm. Muscle fibre area (%) (H), gastrocnemius muscle weight (g) (I), muscle strength (%) (J) and creatine phosphokinase level U/g (K). *P < .05 compared with sham group; ^#^ *P* < .05 compared with PCL conduit group

The effect of the EGCG/PCL conduit on radiation‐induced inflammation was further evaluated using TNF‐α staining. Its expression increased significantly in the PCL conduit and sham groups and decreased significantly in the EGCG/PCL conduit group (Figure 5D‐L,Q). For relative quantification, we performed WB analysis and evaluated the grey value of the bands. Several inflammation‐associated markers were involved, including CD68, TNF‐α and IL‐6. They were all notably downregulated in the EGCG/PCL conduit group and remained highly expressed in the PCL conduit and sham groups (Figure 6A). These results confirm the anti‐inflammatory effect of EGCG in peripheral nerve regeneration.

### 3.8 Effects of the EGCG/PCL conduit on muscle atrophy

The sciatic nerve has important implications for the gastrocnemius muscle. Long‐term radiation therapy induces fibrotic tissue proliferation and nerve encapsulation. Afterwards, nerve compression and degeneration cause severe muscle atrophy. Therefore, sciatic nerve regeneration is associated with morphological and functional recovery of the gastrocnemius muscle. The mean muscle fibre area in the EGCG/PCL conduit group was much larger than in the PCL and sham groups (*P* < .05). Meanwhile, the mean muscle fibre area was also significantly different between the PCL and sham groups (*P* < .05, Figure 6B‐H). Muscle weight and muscle strength increased significantly in the EGCG/PCL conduit group, in comparison with a minor increase in the PCL and sham groups (*P* < .05, Figure 6I,J). In addition, we evaluated creatine phosphokinase (CK) expression and found it to be higher in the EGCG/PCL conduit group at both 6 and 12 weeks after conduit implantation, compared with the other two groups (*P* < .05, Figure 6K). This indicates that EGCG could reverse muscle atrophy to a significant extent in radiation‐induced peripheral neuropathy.

## 4 DISCUSSION

Radiation therapy compromises the immune system and damages functional organs and tissues. With longer survival time, the occurrence of peripheral neuropathy becomes more common after lengthy radiotherapy.^9^ Tissue engineering is widely investigated in regenerative medicine, and this new technique holds great promise for tissue repair and improves therapeutic outcomes.^27^, ^28^, ^29^ Previous reports have discussed surgical intervention and conduit scaffold application for severe peripheral nerve injuries. In addition to structural repair, it is also vital to restore the immune microenvironment surrounding injured nerves. Melatonin‐loaded polycaprolactone scaffolds alleviated immune insult and improved repair of a 15 mm long sciatic nerve insult by regulating the autophagy signalling pathway.^30^ Three‐dimensional nanoceria‐based nerve conduit and nanodiamond channels also contributed to restoration of immune homeostasis by reducing regional oxidative stress and inflammatory reactions in lengthy sciatic nerve defects.^31^, ^32^ Inflammation and oxidative stress are also responsible for radiation‐induced peripheral neuropathy. The onset of inflammation seems to induce the occurrence of oxidative stress and vice versa.^33^ Inflammatory cells such as macrophages can activate ROS and cause oxidative stress. Meanwhile, ROS can also activate intracellular signalling of pro‐inflammatory cues^34^ and both systems take an active part in the pathogenesis of peripheral nerve injury. Damages to nerves immediately induce ROS synthesis and break the protective anti‐oxidant barrier and the balance between pro‐oxidants and anti‐oxidants in the living body. As for inflammation in chronic peripheral neuropathy, macrophages initially not only scavenge myelin debris, but also infiltrate surrounding tissues and structures with lasting effects.^35^ Inflammation and oxidative stress exert a joint effort and exacerbate disease progression. For decades, scientists have been concerned with strategies involving the combination of anti‐oxidant and anti‐inflammatory cues into single agents or biomaterials. Previous studies have introduced many EGCG applications for oxidative stress regulation. Na et al reported that EGCG upregulated Nrf2 expression and activated the downstream extracellular signal‐regulated kinase signalling pathway in epithelial cells.^15^ Krupkova et al discovered that EGCG inhibited p53‐p21 activation and stimulated cell proliferation to protect intervertebral discs from oxidative stress.^36^ Chu et al found that an EGCG‐based collagen scaffold alleviated ROS insults by inhibiting the MAPK p38 signalling pathway.^37^ Qiu et al fabricated a β‐tricalcium phosphate‐loaded conduit by a solvent volatilization method. This scaffold constructed a nutritional microenvironment and reduced oxidant insults while increasing cytoskeletal protein expression.^38^ In this study, we focused on the special function of EGCG in attenuating peripheral neuropathy using an integrated fabrication of the EGCG‐controlled release system. The EGCG/PCL nerve conduit progressively released EGCG for long‐term nerve regeneration. The integrated moulding method has many advantages, such as excellent quality control, controlled drug release and strong mechanical support.

Some anti‐oxidant factors, such as MnSOD, HO‐1 and GCLC, were assayed to evaluate the effects of EGCG on oxidative stress. The EGCG/PCL scaffold upregulated their expression in RSCs and RSMCs compared with the PCL scaffold. Another anti‐oxidant, Nrf2, was also thoroughly investigated. Zhang et al found that Nrf2 knockout mice exhibited relatively lower axon regeneration and myelin debris clearance.^39^ Hsu et al noticed that sesame oil, a natural product, increased GAP‐43 expression in nerve cells and improved recovery after sciatic nerve crush by regulating Nrf2 expression.^40^ Nrf2 may regulate oxidative stress in radiation‐induced peripheral neuropathy. In vitro assays proved Nrf2 expression was upregulated in the EGCG/PCL scaffold, and this was consistent with the suggested changes.

We furthered our study using radiation‐induced rat sciatic neuropathy. A clinically similar radiation‐induced peripheral nerve injury could be created by exposure to 40‐Gy radiation.^4^ Anti‐oxidant proteins, such as nNOS, Nrf2 and MnSOD, were included in analysing the use of EGCG in vivo. Results showed that EGCG significantly downregulated nNOS expression and upregulated Nrf2 and MnSOD expression. In addition, LPO was reduced by EGCG application. Inflammation control was also investigated in this study. Macrophages are closely involved in peripheral nerve regeneration. It was shown that macrophages engulfed broken myelin debris and offered an ideal microenvironment for nerve regrowth at early injury stages.^41^ Another study claimed macrophages accompanied injured sciatic nerve fragmentation after 2 days.^42^ Nevertheless, in later stages, additional efforts were required to terminate the inflammatory effects of macrophages, which harmed long‐term nerve recovery.^43^ Therefore, the relationship between macrophage infiltration and EGCG was investigated. CD68, TNF‐α and IL‐6 expression levels were significantly reduced by the EGCG/PCL conduit, proving its anti‐inflammatory effects.

The gastrocnemius muscle is dominated by the sciatic nerve and can be used as an indicator for nerve recovery.^44^ Muscle weight and strength were highest in the EGCG/PCL conduit group, indicating a significant improvement in muscle fibre restoration. Morphological evaluation showed that muscle fibres displayed a more organized arrangement with EGCG application. In contrast, muscle fibres showed significant shrinkage, smaller size, disorganized structure and reduced total area in the PCL conduit and sham groups. Moreover, CK analysis indicated this muscle atrophy was a chronic process, and it was significant to muscle energy use.^45^ Creatine and adenosine triphosphate are products of the reaction between phosphocreatine and adenosine diphosphate with CK. CK evaluation showed improved results in the EGCG/PCL conduit group. Haramizu et al also confirmed the beneficial effect of catechins in reducing muscle inflammation in an exercise‐oriented muscle damage model.^46^ These results indicate successful reinnervation of the gastrocnemius muscle fibre with EGCG‐controlled release. In this study, no rats experienced exceptional pain. There are several novel aspects of this study. First, as an excellent natural drug, EGCG was used for peripheral nerve injury repair. However, this is the first time that EGCG has been fabricated as a nerve conduit with PCL to repair severe radiation‐induced neuropathy. In addition, it is the first time that EGCG has been confirmed to reduce gastrocnemius muscle atrophy. The gastrocnemius muscle is an effector of the sciatic nerve and is important to animal activity. It is also the first time that Nrf2 signalling has been confirmed to be related to EGCG in peripheral nerve regeneration.

## 5 CONCLUSION

The EGCG‐modified PCL conduit was characterized by controlled release and nutrition exchange. An implantable drug carrier released a small and beneficial dosage at intervals over an extended period. From our results, the EGCG/PCL scaffold induced a lasting effect on the anti‐oxidant and anti‐inflammatory functions for long‐term recovery. In addition, satisfactory nerve regeneration was confirmed in functional and electrophysiological experiments, facilitated by the proliferative induction of EGCG and a porous structure for nutrition exchanges. Therefore, the EGCG‐modified PCL nerve conduit improved peripheral nerve regeneration and gastrocnemius muscle restoration over a lengthy regenerative process. This finding may provide insights into research on polyphenols for nerve regeneration.

## CONFLICT OF INTEREST

The authors declare no conflict of interest.

## AUTHORS' CONTRIBUTIONS

W. Yuan, C. Fan and Y. Ouyang conceived the initial idea, the conceptualization and the study design, and participated in the data extraction and analysis. Y. Qian, Z. Yao, X. Wang, Y. Cheng, Z. Fang, Y. Ouyang, W. Yuan and C. Fan participated in its design, searched databases and extracted the studies. Y. Qian prepared the draft. W. Yuan, C. Fan and Y. Ouyang revised the manuscript. All authors read and approved the final manuscript.

## Data Availability

The data that support the findings of this study are available from the corresponding author upon reasonable request.

## References

[1] Okuhara Y , Shinomiya R , Peng F , et al. Direct effect of radiation on the peripheral nerve in a rat model. J Plast Surg Hand Surg. 2014;48(4):276‐280. 24479792 10.3109/2000656X.2014.882343

[2] Etminan M , Brophy JM , Samii A . Oral fluoroquinolone use and risk of peripheral neuropathy: a pharmacoepidemiologic study. Neurology. 2014;83(14):1261‐1263. 25150290 10.1212/WNL.0000000000000846

[3] Alport AR , Sander HW . Clinical approach to peripheral neuropathy: anatomic localization and diagnostic testing. Continuum. 2012;18(1):13‐38. 22810068 10.1212/01.CON.0000411546.13207.b1

[4] Liao C , Zheng R , Wei C , et al. Tissue‐engineered conduit promotes sciatic nerve regeneration following radiation‐induced injury as monitored by magnetic resonance imaging. Magn Reson Imaging. 2016;34(4):515‐523. 26686023 10.1016/j.mri.2015.12.004

[5] Lundborg G , Dahlin LB , Danielsen N , et al. Nerve regeneration in silicone chambers: influence of gap length and of distal stump components. Exp Neurol. 1982;76(2):361‐375. 7095058 10.1016/0014-4886(82)90215-1

[6] den Dunnen WF , van der Lei B , Schakenraad JM , et al. Poly(DL‐lactide‐epsilon‐caprolactone) nerve guides perform better than autologous nerve grafts. Microsurgery. 1996;17(7):348‐357. 9379881 10.1002/(SICI)1098-2752(1996)17:7<348::AID-MICR2>3.0.CO;2-C

[7] Weber RA , Breidenbach WC , Brown RE , Jabaley ME , Mass DP . A randomized prospective study of polyglycolic acid conduits for digital nerve reconstruction in humans. Plast Reconstr Surg. 2000; 106(5): 1036‐1045; discussion 1046–1048. 11039375 10.1097/00006534-200010000-00013

[8] Farrow A , Morrison R , Pickersgill T , Currie R , Hammersley N . Transient femoral neuropathy after harvest of bone from the iliac crest. Br J Oral Maxillofac Surg. 2004;42(6):572‐574. 15544891 10.1016/j.bjoms.2004.06.014

[9] Pradat PF , Delanian S . Late radiation injury to peripheral nerves. Handb Clin Neurol. 2013;115:743‐758. 23931813 10.1016/B978-0-444-52902-2.00043-6

[10] Denham JW , Hauer‐Jensen M . The radiotherapeutic injury–a complex 'wound'. Radiother Oncol. 2002;63:129‐145. 12063002 10.1016/s0167-8140(02)00060-9

[11] Sandireddy R , Yerra VG , Areti A , Komirishetty P , Kumar A . Neuroinflammation and oxidative stress in diabetic neuropathy: futuristic strategies based on these targets. Int J Endocrinol. 2014;2014:674987. 24883061 10.1155/2014/674987 PMC4021687

[12] Cooper R . Green tea and theanine: health benefits. Int J Food Sci Nutr. 2012;63(Suppl 1):90‐97. 10.3109/09637486.2011.629180 22039897

[13] Han SY , Kim E , Hwang K , et al. Cytoprotective Effect of Epigallocatechin Gallate (EGCG)‐5'‐O‐α‐Glucopyranoside, a Novel EGCG Derivative. Int J Mol Sci. 2018;19(5):1466. 10.3390/ijms19051466 PMC5983637 29762498

[14] Wei IH , Tu HC , Huang CC , Tsai MH , Tseng CY , Shieh JY . (‐)‐Epigallocatechin gallate attenuates NADPH‐d/nNOS expression in motor neurons of rats following peripheral nerve injury. BMC Neurosci. 2010;12:52. 10.1186/1471-2202-12-52 PMC3121620 21627848

[15] Na HK , Kim EH , Jung JH , Lee HH , Hyun JW , Surh YJ . (−)‐Epigallocatechin gallate induces Nrf2‐mediated antioxidant enzyme expression via activation of PI3K and ERK in human mammary epithelial cells. Arch Biochem Biophys. 2008;476(2):171‐177. 18424257 10.1016/j.abb.2008.04.003

[16] Barbarisi M , Marino G , Armenia E , et al. Use of polycaprolactone (PCL) as scaffolds for the regeneration of nerve tissue. J Biomed Mater Res A. 2015;103(5):1755‐1760. 25202882 10.1002/jbm.a.35318

[17] Sun G , Wei D , Liu X , et al. Novel biodegradable electrospun nanofibrous P(DLLA‐CL) balloons for the treatment of vertebral compression fractures. Nanomedicine. 2013;9(6):829‐838. 23318398 10.1016/j.nano.2012.12.003

[18] Xu N , Ye X , Wei D , et al. 3D artificial bones for bone repair prepared by computed tomography‐guided fused deposition modeling for bone repair. ACS Appl Mater Interfaces. 2014;6(17):14952‐14963. 25133309 10.1021/am502716t

[19] Liu X , Wei D , Zhong J , et al. Electrospun Nanofibrous P(DLLA‐CL) Balloons as Calcium Phosphate Cement Filled Containers for Bone Repair: in Vitro and in Vivo Studies. ACS Appl Mater Interfaces. 2015;7(33):18540‐18552. 26258872 10.1021/acsami.5b04868

[20] Zhong J , Zhang H , Yan J , Gong X . Effect of nanofiber orientation of electrospun nanofibrous scaffolds on cell growth and elastin expression of muscle cells. Colloids Surf B Biointerfaces. 2015;136:772‐778. 26520049 10.1016/j.colsurfb.2015.10.017

[21] Reid AJ , de Luca AC , Faroni A , et al. Long term peripheral nerve regeneration using a novel PCL nerve conduit. Neurosci Lett. 2013;544:125‐130. 23583695 10.1016/j.neulet.2013.04.001

[22] Neal RA , Lenz SM , Wang T , et al. Laminin‐ and basement membrane‐polycaprolactone blend nanofibers as a scaffold for regenerative medicine. Nanomater Environ. 2014;2(1):1‐12. 27570767 10.2478/nanome-2014-0001 PMC4999083

[23] Saderi N , Rajabi M , Akbari B , Firouzi M , Hassannejad Z . Fabrication and characterization of gold nanoparticle‐doped electrospun PCL/chitosan nanofibrous scaffolds for nerve tissue engineering. J Mater Sci Mater Med. 2018;29(9):134. 30120577 10.1007/s10856-018-6144-3

[24] Qian Y , Song J , Zheng W , et al. 3D manufacture of gold nanocomposite channels facilitates neural differentiation and regeneration. Adv Funct Mater. 2018;28:1707077.

[25] Qian Y , Song J , Zhao X , et al. 3D Fabrication with Integration Molding of a Graphene Oxide/Polycaprolactone Nanoscaffold for Neurite Regeneration and Angiogenesis. Adv Sci. 2018;5(4):1700499. 10.1002/advs.201700499 PMC5908351 29721407

[26] Qian Y , Zhao X , Han Q , Chen W , Li H , Yuan W . An integrated multi‐layer 3D‐fabrication of PDA/RGD coated graphene loaded PCL nanoscaffold for peripheral nerve restoration. Nat Commun. 2018;9(1):323. 29358641 10.1038/s41467-017-02598-7 PMC5778129

[27] Dzobo K , Thomford NE , Senthebane DA , et al. Advances in regenerative medicine and tissue engineering: innovation and transformation of medicine. Stem Cells Int. 2018;30(2018):2495848. 10.1155/2018/2495848 PMC6091336 30154861

[28] Fricain JC , De Olivera H , Devillard R , et al. 3D bioprinting in regenerative medicine and tissue engineering. Med Sci. 2017;33(1):52‐59. 10.1051/medsci/20173301009 28120756

[29] Gomes ME , Rodrigues MT , Domingues RMA , Reis RL . Tissue Engineering and Regenerative Medicine: New Trends and Directions‐A Year in Review. Tissue Eng Part B Rev. 2017;23(3):211‐224. 28457175 10.1089/ten.TEB.2017.0081

[30] Qian Y , Han Q , Zhao X , et al. 3D melatonin nerve scaffold reduces oxidative stress and inflammation and increases autophagy in peripheral nerve regeneration. J Pineal Res. 2018;65(4):e12516. 29935084 10.1111/jpi.12516

[31] Qian Y , Han Q , Zhao X , Li H , Yuan WE , Fan C . Asymmetrical 3D Nanoceria Channel for Severe Neurological Defect Regeneration. iScience. 2019;12:216‐231. 30703735 10.1016/j.isci.2019.01.013 PMC6354782

[32] Qian Y , Cheng Y , Ouyang Y , Yuan WE , Fan C . Multilayered spraying and gradient dotting of nanodiamond–polycaprolactone guidance channels for restoration of immune homeostasis. NPG Asia Mater. 2019;11:36.

[33] Dandekar A , Mendez R , Zhang K . Cross talk between ER stress, oxidative stress, and inflammation in health and disease. Methods Mol Biol. 2015;1292:205‐214. 25804758 10.1007/978-1-4939-2522-3_15

[34] Cros J , Cagnard N , Woollard K , et al. Human CD14 monocytes patrol and sense nucleic acids and viruses via TLR7 and TLR8 receptors. Immunity. 2010;33(3):375‐386. 20832340 10.1016/j.immuni.2010.08.012 PMC3063338

[35] Klein D , Martini R . Myelin and macrophages in the PNS: An intimate relationship in trauma and disease. Brain Res. 2016;1641(Pt A):130‐138. 26631844 10.1016/j.brainres.2015.11.033

[36] Krupkova O , Sekiguchi M , Klasen J , et al. Epigallocatechin 3‐gallate suppresses interleukin‐1β‐induced inflammatory responses in intervertebral disc cells in vitro and reduces radiculopathic pain in rats. Eur Cell Mater. 2014;28:372‐386. 25422948 10.22203/ecm.v028a26

[37] Chu C , Deng J , Cao C , Man Y , Qu Y . Evaluation of epigallocatechin‐3‐gallate modified collagen membrane and concerns on Schwann cells. Biomed Res Int. 2017;2017:9641801. 28894753 10.1155/2017/9641801 PMC5574217

[38] Qiu T , Yin Y , Li B , et al. PDLLA/PRGD/β‐TCP conduits build the neurotrophin‐rich microenvironment suppressing the oxidative stress and promoting the sciatic nerve regeneration. J Biomed Mater Res A. 2014;102(10):3734‐3743. 24408878 10.1002/jbm.a.35078

[39] Zhang L , Johnson D , Johnson JA . Deletion of Nrf2 impairs functional recovery, reduces clearance of myelin debris and decreases axonal remyelination after peripheral nerve injury. Neurobiol Dis. 2013;54:329‐338. 23328769 10.1016/j.nbd.2013.01.003 PMC3628945

[40] Hsu CC , Huang HC , Wu PT , Tai TW , Jou IM . Sesame oil improves functional recovery by attenuating nerve oxidative stress in a mouse model of acute peripheral nerve injury: role of Nrf‐2. J Nutr Biochem. 2016;38:102‐106. 27732910 10.1016/j.jnutbio.2016.09.003

[41] Conforti L , Gilley J , Coleman MP . Wallerian degeneration: an emerging axon death pathway linking injury and disease. Nat Rev Neurosci. 2014;15(6):394‐409. 24840802 10.1038/nrn3680

[42] Mietto BS , Mostacada K , Martinez AM . Neurotrauma and Inflammation: CNS and PNS Responses. Mediators Inflamm. 2015;2015:251204. 25918475 10.1155/2015/251204 PMC4397002

[43] Sun X , Bai Y , Zhai H , et al. Devising micro/nano‐architectures in multi‐channel nerve conduits towards a pro‐regenerative matrix for the repair of spinal cord injury. Acta Biomater. 2019;86:194‐206. 30586646 10.1016/j.actbio.2018.12.032

[44] Asano K , Nakano T , Tokutake K , et al. Muscle spindle reinnervation using transplanted embryonic dorsal root ganglion cells after peripheral nerve transection in rats. Cell Prolif. 2019;52(5):e12660. 31264327 10.1111/cpr.12660 PMC6797520

[45] Erickson‐Viitanen S , Geiger P , Yang WC , Bessman SP . The creatine‐creatine phosphate shuttle for energy transport — compartmentation of creatine phosphokinase in muscle. Adv Exp Med Biol. 1982;151:115‐125. 6217725 10.1007/978-1-4684-4259-5_17

[46] Haramizu S , Ota N , Hase T , Murase T . Catechins suppress muscle inflammation and hasten performance recovery after exercise. Med Sci Sports Exerc. 2013;45(9):1694‐1702. 23470311 10.1249/MSS.0b013e31828de99f

