# Supplementary material for: (‐)‐Epigallocatechin gallate‐loaded polycaprolactone scaffolds fabricated using a 3D integrated moulding method alleviate immune stress and induce neurogenesis
Source: Cell Prolif. 2019 Nov 20;53(1):e12730. doi: 10.1111/cpr.12730 (PMC6985678; doi:10.1111/cpr.12730)
Supplement: Supplementary file 1 [file CPR-53-e12730-s001.docx]

**Supporting Information**


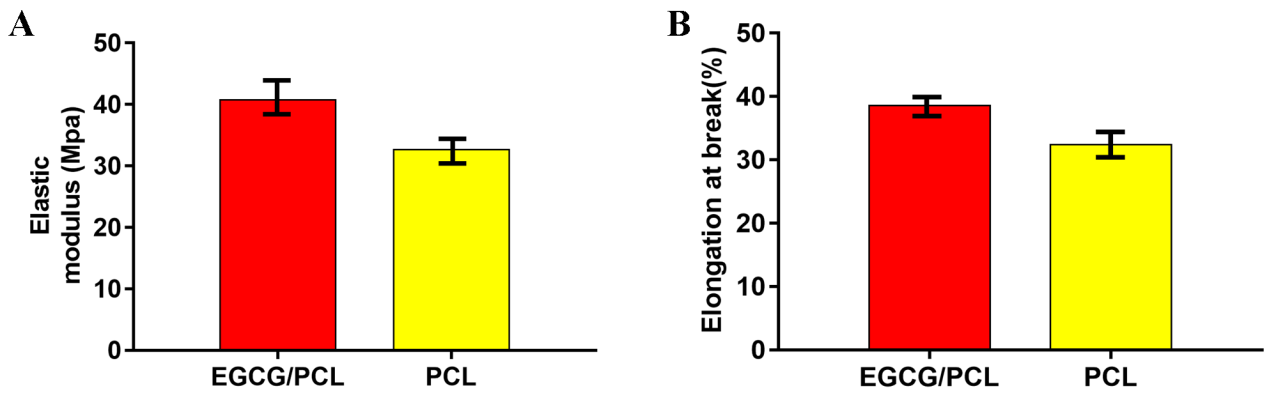


**Fig. S1.** Mechanical properties like elastic modulus (A) and elongation at break (B) of EGCG/PCL and PCL scaffolds.


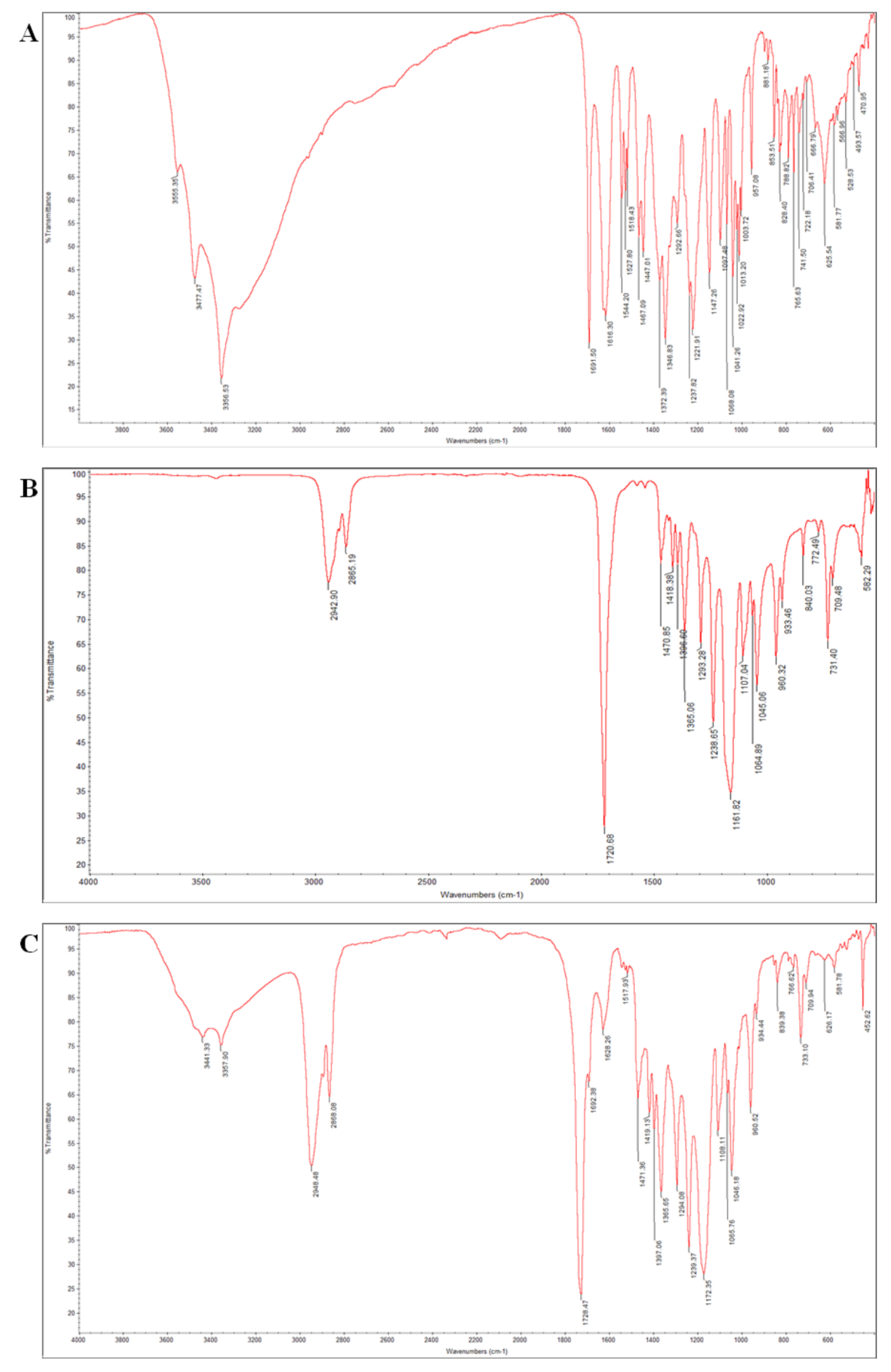


**Fig. S2.** FTIR assay for evaluation of EGCG-PCL interaction in the scaffold. A. EGCG; B. PCL scaffold; C. EGCG/PCL scaffold.


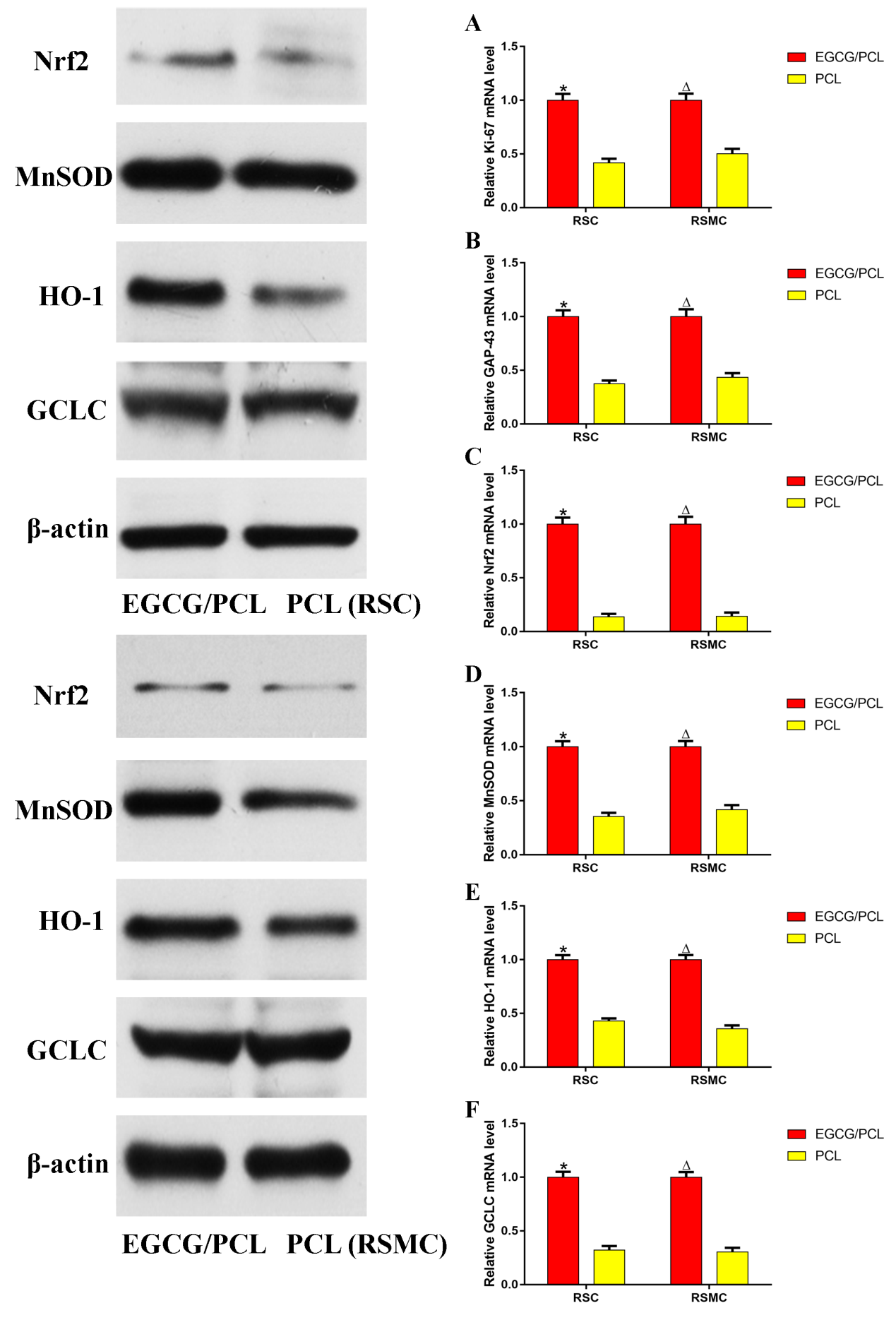


**Fig. S3.** WB and qPCR for Ki-67 (A), GAP-43 (B), Nrf2 (C), MnSOD (D), HO-1 (E), and GCLC (F) expression of RSCs and RSMCs on EGCG/PCL and PCL scaffolds. *p<0.05 compared with PCL scaffolds (RSCs); Δp<0.05 compared with PCL scaffolds (RSMCs).


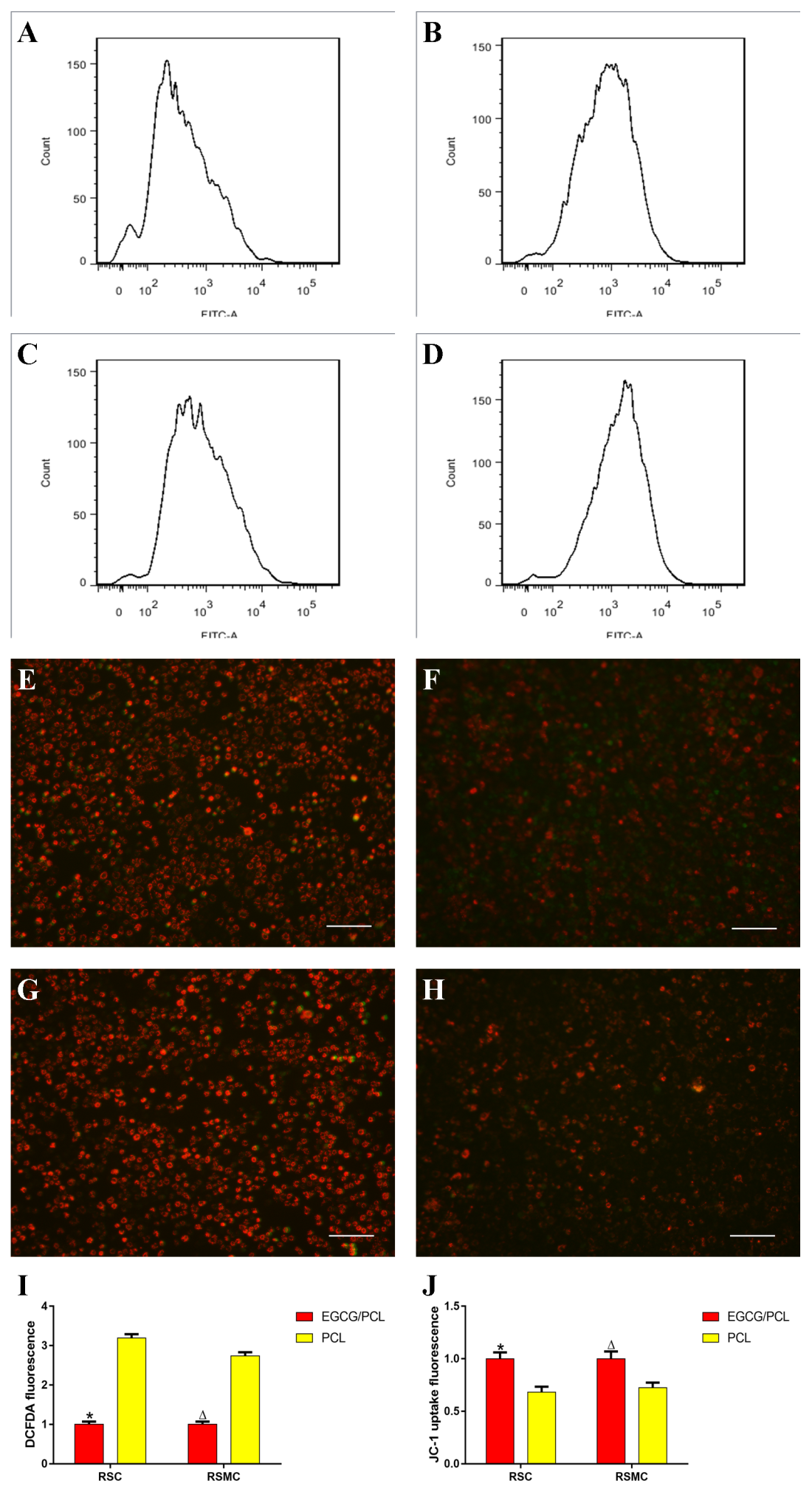


**Fig. S4.** Flow cytometry assay for intracellular ROS evaluation with DCFDA staining for RSCs on EGCG/PCL scaffolds (A) and PCL scaffolds (B), and RSMCs on EGCG/PCL scaffolds (C) and PCL scaffolds (D). The fluorescent images for mitochondrial membrane potential by JC-1 staining. JC-1 uptake from RSCs on EGCG/PCL scaffolds (E) and PCL scaffolds (F), and RSMCs on EGCG/PCL scaffolds (G) and PCL scaffolds (H). The relative fluorescent expression levels for ROS expression and JC-1 staining between two groups are displayed in (I) and (J). *p<0.05 compared with PCL scaffold (RSCs); Δp<0.05 compared with PCL scaffolds (RSMCs). The scale bar is 100 μm.


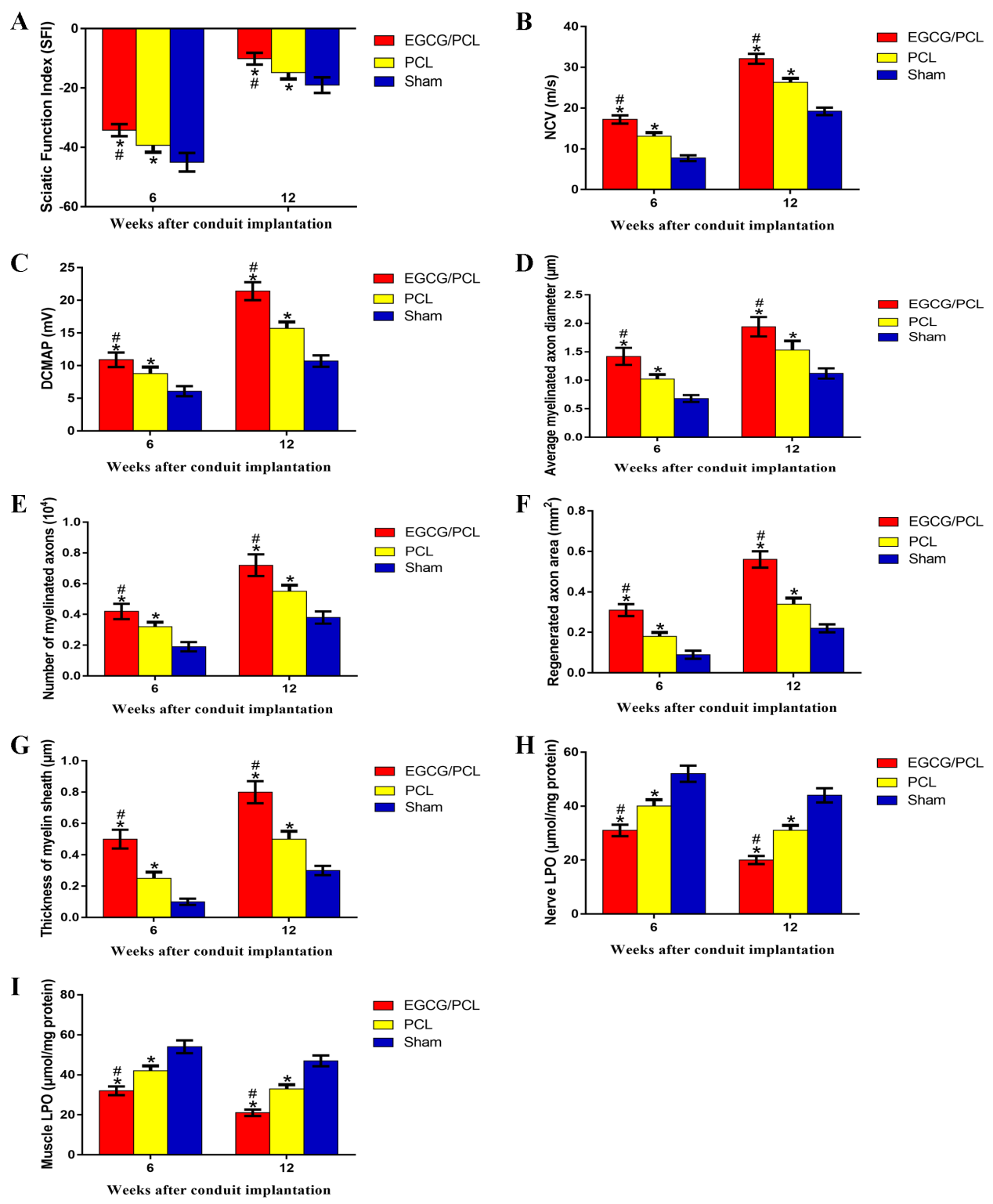


**Fig. S5.** Functional, morphological and electrophysiological evaluation for EGCG/PCL conduit group, PCL conduit group and sham group at 6 and 12 weeks after conduit implantation. Sciatic Function Index (SFI) (A), NCV (m/s) (B), DCMAP (mV) (C), Average myelinated axon diameter (μm) (D), Number of myelinated axons (10^4^) (E), Regenerated axon area (mm^2^) (F), Thickness of myelin sheath (μm) (G), Nerve LPO level (μmol/mg protein) (H), Muscle LPO level (μmol/mg protein) (I). *p<0.05 compared with sham group; #p<0.05 compared with PCL group.


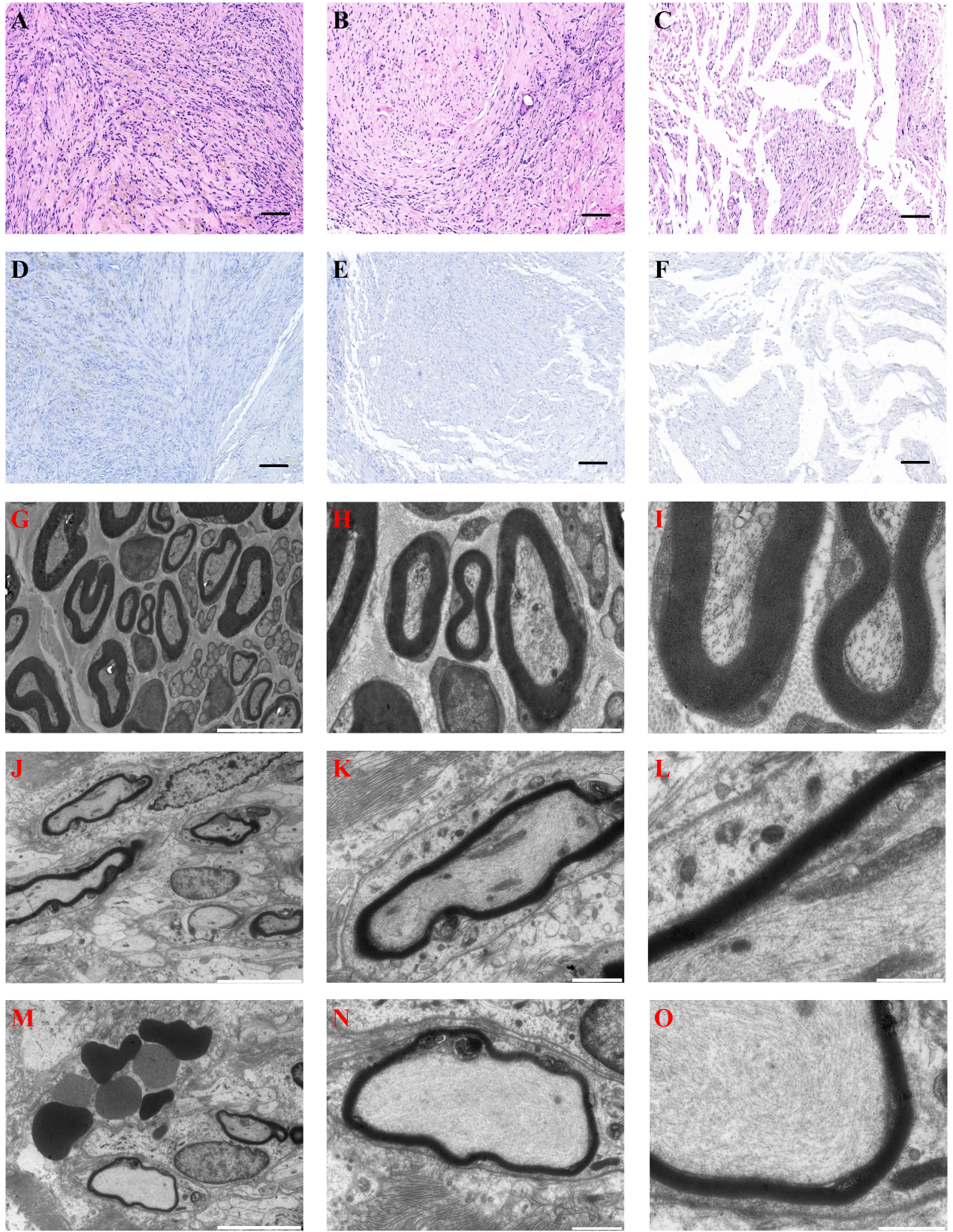


**Fig. S6.** HE staining and TB staining for regenerated sciatic nerves from three groups.

Fig. S6A (HE staining) and Fig. S6D (TB staining) represent EGCG/PCL conduit group. Fig. S6B (HE staining) and Fig. S6E (TB staining) represent PCL conduit group. Fig. S6C (HE staining) and Fig. S6F (TB staining) represent sham group. All sciatic nerves samples were dissected from the parts in the conduit or middle portion from sham group. The scale bar is 100 μm. Transmission electron microscopy for transverse sections of regenerated nerves in three groups. EGCG/PCL (G, H, I); PCL (J, K, L); Sham (M, N, O). The scale bar is 10 μm (G, J, M), 2 μm (H, K, N) and 1 μm (I, L, O) respectively.

**Table S1.** Primer sequences in RT-PCR assay

| Primer Name | Left | Right |
| --- | --- | --- |
| Nrf2 | CTCTCTGAACTTCTGGGCGG | ACGTTGCCATCTCTGGTCTG |
| MnSOD | CACCGAGGAGAAGTACCACG | TGGGTTCTCCACCACCCTTA |
| HO-1 | TTAAGCTGGTGATGGCCTCC | GTGGGGCATAGACTGGGTTC |
| GCLC | TACCGAGGCTACGTGTCAGA | GCTCGTAGATCTCCGTGTCG |
| Ki-67 | ACAGGGCTTAGGAAACAGTCC | GGGTTCTAACTGGTCTTCCTGG |
| GAP-43 | ACCTAAGGAAAGTGCCCGAC | GGAGAGACAGGGTTCAGGTG |
